# Pre-treatment of Single and Double Antiplatelet and Anticoagulant With Intravenous Thrombolysis for Older Adults With Acute Ischemic Stroke: The TTT-AIS Experience

**DOI:** 10.3389/fneur.2021.628077

**Published:** 2021-02-22

**Authors:** Sheng-Feng Lin, Han-Hwa Hu, Bo-Lin Ho, Chih-Hung Chen, Lung Chan, Huey-Juan Lin, Yu Sun, Yung-Yang Lin, Po-Lin Chen, Shinn-Kuang Lin, Cheng-Yu Wei, Yu-Te Lin, Jiunn-Tay Lee, A-Ching Chao, Taiwan Thrombolytic Therapy for Acute Ischemic Stroke (TTT-AIS) Study Group

**Affiliations:** ^1^School of Public Health, College of Public Health, Taipei Medical University, Taipei, Taiwan; ^2^Department of Critical Care Medicine, Taipei Medical University Hospital, Taipei, Taiwan; ^3^Department of Clinical Pathology, Taipei Medical University, Taipei, Taiwan; ^4^Beijing Tiantan Hospital, Capital Medical University, Beijing, China; Advanced Innovation Center for Human Brain Protection, Capital Medical University, Beijing, China; ^5^Department of Neurology, Taipei Medical University-Shaung Ho Hospital, Taipei, Taiwan; ^6^Department of Neurology, Kaohsiung Medical University Hospital, Kaohsiung, Taiwan; ^7^Department of Neurology, Kaohsiung Medical University, Kaohsiung, Taiwan; ^8^Department of Neurology, National Cheng Kung University Hospital, Tainan, Taiwan; ^9^Department of Neurology, National Cheng Kung University, Tainan, Taiwan; ^10^Department of Neurology, Chi Mei Medical Center, Tainan, Taiwan; ^11^Department of Neurology, En Chu Kong Hospital, New Taipei City, Taiwan; ^12^Department of Neurology, Taipei Veterans General Hospital, Taipei, Taiwan; ^13^Department of Neurology, Taichung Veterans General Hospital, Taichung, Taiwan; ^14^Stroke Center and Department of Neurology, Taipei Tzu Chi Hospital, Buddhist Tzu Chi Medical Foundation, Taipei, Taiwan; ^15^Department of Neurology, Show Chwan Memorial Hospital, Changhua, Taiwan; ^16^Division of Neurology, Department of Medicine, Kaohsiung Veterans General Hospital, Kaohsiung, Taiwan; ^17^Department of Neurology, National Defense Medical Center, Tri-Service General Hospital, Taipei, Taiwan

**Keywords:** aspirin, clopidogrel, stroke, intracranial hemorrhage, intravenous thrombolysis

## Abstract

**Background:** This study aimed to investigate the safety and efficacy of single antiplatelet, anticoagulant and Dual Antiplatelet pre-treatment (DAPP) in older, moderate to high severity acute ischemic stroke patients treated with intravenous thrombolysis (IVT).

**Methods:** A prospective cohort study was conducted to monitor the development of symptomatic intracranial hemorrhage (SICH) and functional outcomes at 90 days. Two different dosages of alteplase were used for IVT. Logistic regression models were used for analysis of the safety and efficacy outcomes.

**Results:** A total of 1,156 patients were enrolled and categorized into six groups based on their pre-treatment medications: (1) aspirin (n = 213), (2) clopidogrel (*n* = 37), (3) DAPP of aspirin + clopidogrel (*n*= 27), (4) warfarin (*n* = 44), (5) any of the above pre-medications (*n* = 331), and (6) none of these medications as controls (*n* = 825). The DAPP group showed significantly increased SICH by the NINDS (adjusted OR: 4.90, 95% CI 1.28–18.69) and the ECASS II (adjusted OR: 5.09, 95% CI: 1.01–25.68) standards. The aspirin group was found to significantly improve the favorable functional outcome of the modified Rankin Scale (mRS) of 0–1 (adjusted OR: 1.91, 95% CI, 1.31.2.78), but no significance for mRS of 0–2 (adjusted OR: 1.39, 95% CI, 0.97–1.99). The DAPP group also significantly increased mortality (adjusted OR: 4.75, 95% CI: 1.77–12.72). A significant interaction between different dosages for IVT and the functional status was noted. Compared to standard dose, the DAPP group showed higher proportions of disability and mortality with low dose of IVT.

**Conclusion:** For older adults with higher baseline severity of acute ischemic stroke, DAPP may increase the risk of SICH and mortality post IVT. However, DAPP is still not an indication to withdraw IVT and to prescribe low-dose IVT for older adults.

## Introduction

A *post hoc* analysis from the randomized controlled trial (RCT) of Enhanced Control of Hypertension and Thrombolysis Stroke (ENCHANTED) Study (1) indicated a significant interaction between the different doses of intravenous thrombolysis (IVT) and pre-treatment of antiplatelet (2). Compared to the standard dose of IVT with alteplase, the low-dose group showed increased favorable functional outcome after the pre-treatment with antiplatelets (2). They found that, the pre-treatment of antiplatelets with IVT revealed borderline significance for increased Symptomatic Intracranial Hemorrhage (SICH) (2) according to terms of the Safe Implementation of Thrombolysis in Stroke- Monitoring Study (SITS-MOST) criteria (3).

Previous studies on patients with acute ischemic stroke who were treated with IVT found a 2-fold risk of increased SICH with a single antiplatelet pre-treatment (4–6), and 4- to 9-fold increased risk with dual antiplatelet pre-treatment (DAPP) (4–6). This extremely high risk of increased SICH with DAPP was likely caused by selection bias. Two recent studies by Tsivgoulis et al. (7, 8) employed propensity score matching (PSM) to control the imbalance of the confounders between both the groups, with and without DAPP. Their results suggest that DAPP caused no significant increase in SICH by most standards except of the SITS-MOST criteria, and no significant improvement in the Favorable Functional Outcome (FFO) (7, 8). A recent pooled analysis study showed similar findings at first (9); however, a recent letter to this study revealed that the pooled results were biased by duplicate data and disproved the major findings (10). They indicated that DAPP significantly increased SICH by deleting duplicate data (10). Thereafter, some issues remain to be answered. First, studies employing the PSM method focused on mild ischemic stroke severity with a National Institute of Health Stroke Scale (NIHSS) score of < 10 (7, 8). Second, there was a high heterogeneity in the definition of DAPP (including both aspirin + dipyridamole and aspirin + clopidogrel) and SICH standards in the pooled analysis (9). Third, we considered that older patients were more susceptible to bleeding with DAPP.

The aim of this study was to investigate whether pre-treatment with single antiplatelet, warfarin, and DAPP for acute ischemic stroke patients who were treated with IVT with the following characteristics: (1) older age, (2) moderate to high severity with high NIHSS score at baseline, and (3) the low-dose alteplase, imposed changed risk of SICH and the global functional outcomes.

## Methods

### Study Design and Patients

The Taiwan Thrombolytic Therapy for Acute Ischemic Stroke (TTT-AIS) study was a multicenter, prospective cohort design, which was conducted between December 1, 2004 and December 31, 2016, throughout all regions in Taiwan. A detailed description of the data collection of TTT-AIS has been published previously (11–13). The TTT-AIS data sets include demographic characteristics, previous medical history, such as hypertension, diabetes mellitus, hyperlipidemia, atrial fibrillation, and alcoholism; time duration between stroke onset and IV thrombolysis; NIHSS at baseline; blood pressure at initial presentation; alteplase dose for IVT; levels of glucose, prothrombin time or international normalized ratio (INR), and activated partial thromboplastin time (aPTT) before IVT. All patients underwent brain computed tomography (CT) scans prior to IVT, and another brain CT scan was conducted within 24–36 h post IVT. The indications and contraindications for IVT were referred to the SITS-MOST (13) study except an upper age limit of 80 years. Patients treated with oral anticoagulant (including warfarin) with INR >1.7 was excluded for IVT.

Patients were eligible for enrollment if they met the following inclusion criteria: (1) age ≥ 60 years, (2) clinical diagnosis of acute ischemic stroke with treatment of intravenous thrombolysis within 3 h of stroke onset, and (3) information on the antiplatelets or anticoagulants used before the stroke onset between December 1, 2004 and December 31, 2016. Based on this data, we categorized these patients into the 6 premedication groups: (1) aspirin use (ASA), (2) clopidogrel or ticlopidine use (P2Y12), (3) dual antiplatelets of aspirin and clopidogrel use (DAPP), (4) warfarin use (WAR), (5) any antiplatelet or anticoagulant use (Any AP/AC), and (6) no use of antiplatelets or anticoagulants (no AP/AC). This study was approved by the Institutional Review Board of Kaohsiung Medical University (reference number: KMUH-IRB-20140305). Informed consent was obtained from the patients prior to their inclusion in the study.

### Outcome Measures

For the safety outcome, two standards for SICH were used: (1) the National Institute of Neurological Disorders and Stroke (NINDS) criteria (14); intracranial hemorrhage with an increase of NIHSS ≥ 1 point or death within 36 h, (2) the European-Australasian Acute Stroke Study II (ECASS II); (14) intracranial hemorrhage with deterioration of NIHSS ≥ 4 point or death compared with baseline NIHSS within 36 h. Functional outcomes were assessed according to the modified Rankin Scale (mRS) (15). For the efficacy outcome, two definitions of better outcomes were employed: the favorable functional outcome (FFO) was defined as mRS of 0–1 at 90 days, and functional independence (FI) was taken as mRS of 0–2 at 90 days. Mortality (mRS of 6) at 90 days was also assessed.

### Statistical Analysis

Continuous variables were compared using Student's *t*-test, while discrete variables were compared using the Chi-square or Fisher exact test. First, the associations among no AP/AC, prior AP/AC, and DAPP use on global functional outcome were analyzed using ordinal logistic regression analysis. Second, logistic regression was employed to estimate the OR (odds ratio) for outcome measures of SICH within 36 h of stroke onset and FFO, FI, and mortality at 90 days. The AP or AC naïve (No AP/AC) group was used as the control group. In addition, multivariate regression models were applied to adjust for the characteristic difference between the premedication group and no AP/AC group. Statistical significance was defined as (two-tailed) *P-*value < 0.05. All analysis were performed with the SAS 9.4 software (SAS Institute, Cary, NC, USA).

## Results

### Characteristics of Enrolled Patients

A total of 1,156 patients aged ≥ 60 years were enrolled in this study (Table 1). Of these, 825 patients were categorized as having no AP/AC, and 331 were classified as having AP/AC. Of the any AP/ AC group, 213 patients were pre-treated with ASA, 37 with P2Y12, 27 with DAPP of ASA and clopidogrel, 44 with AC of warfarin, and 10 with other antiplatelets. Of the 10 patients, four patients were treated with dipyridamole and six stroke patients were with cilostazol, respectively (Supplementary Table 1). Of each group, the average age was in the range of 74–77 years. Around 40% of the patients were female. Overall, age and sex distribution in the six groups were in fact homogenous without significant differences. Regarding medical comorbidities, the ASA group had higher proportions of hypertension and diabetes, and the WAR group had higher proportions of atrial fibrillation. Both the P2Y12 and DAPP groups showed no significant difference in medical comorbidity compared to the no AP/AC group. Laboratory tests of glucose, INR, aPTT, and blood pressure showed no statistical significance. Moreover, patients in all six groups had moderate to high severity of acute ischemic stroke at baseline (mean NIHSS between 13 and 16), and 70% of patients were treated with low-dose alteplase for IVT. The onset-to-needle time was approximately 120 min for each group.

**Table 1:** Characteristics of patients receiving antiplatelets and anticoagulants before intravenous thrombolysis (Total *N* = 1,156).

	**ASA (*n* = 213)**	**P2Y12 (*n* = 37)**	**DAPP (*n* = 27)**	**WAR (*n* = 44)**	**Any AP/AC (*n* = 331)**	**No AP/AC (*n* = 825)**
Age (years)	74.5 ± 7.9	76.6 ± 10.2	77.5 ± 7.5	74.3 ± 7.8	74.9 ± 8.3	74.9 ± 8.6
**Age groups (years)**						
60–69 years	30.5% (65/213)	32.4% (12/37)	18.5% (5/27)	34.1% (15/44)	30.8% (102/331)	30.2% (249/825)
70–79 years	41.3% (88/213)	27.0% (10/37)	44.4% (12/27)	36.4% (16/44)	39.0% (129/331)	39.6% (327/825)
80–89 years	24.4% (52/213)	29.7% (11/37)	33.3% (9/27)	27.3% (12/44)	25.7% (85/331)	24.9% (205/825)
≥90 years	3.8% (8/213)	10.8% (4/37)	3.7% (1/27)	2.3% (1/44)	4.5% (15/331)	5.3% (44/825)
Female sex (%)	39.0% (83/213)	32.4% (12/37)	48.2% (13/27)	43.2% (19/44)	39.9% (132/331)	39.5% (326/825)
**Comorbidity (%)**						
Hypertension	83.1% (177/213)^**^	83.8% (31/37)	59.3% (16/27)	65.9% (29/44)	78.9% (261/331)	74.4% (614/825)
Diabetes	44.6% (95/213)^***^	40.5% (15/37)	48.2% (13/27)	38.6% (17/44)	43.8% (145/331)^***^	30.6% (252/825)
Hyperlipidemia	27.2% (58/213)^*^	29.7% (11/37)	29.6% (8/27)	25.0% (11/44)	27.2% (90/331)^**^	35.9% (296/825)
Atrial fibrillation	46.6% (90/193)	45.7% (16/35)	53.9% (14/26)	90.2% (37/41)^***^	52.5% (159/303)^***^	40.0% (303/757)
Alcoholism	5.6% (12/213)	8.1% (3/37)	11.1% (3/27)	4.6% (2/44)	6.3% (21/331)	4.9% (40/825)
Glucose (mg/dl)	151.8 ± 53.9	156.0 ± 49.0	183.7 ± 95.5	141.9 ± 52.1	153.2 ± 57.7	148.4 ± 69.8
Prothrombin time (INR)	1.02 ± 0.11	1.00 ± 0.07	0.99 ± 0.11	1.13 ± 0.21^**^	1.03 ± 0.13	1.02 ± 0.10
aPTT	28.2 ± 4.8	28.1 ± 6.2	27.6 ± 4.0	29.8 ± 4.2	28.3 ± 4.8	29.7 ± 13.9
Systolic BP (mmHg)	158.2 ± 28.2^**^	159.1 ± 27.4	162.9 ± 29.5	152.4 ± 31.0	157.7 ± 28.5^***^	164.9 ± 29.6
Diastolic BP (mmHg)	88.9 ± 19.1^*^	87.9 ± 18.5	89.4 ± 18.3	88.9 ± 21.3	88.8 ± 19.0^*^	92.0 ± 19.2
Baseline NIHSS	13.1 ± 6.6	14.5 ± 7.0	15.5 ± 5.1	15.9 ± 7.7	13.8 ± 6.8	13.9 ± 7.2
Alteplase dose (mg/kg)	0.77 ± 0.15^**^	0.74 ± 0.17	0.80 ± 0.15	0.78 ± 0.15	0.77 ± 0.15^***^	0.81 ± 0.15
Standard dose	27.2% (58/213)	29.7% (11/37)	25.9% (7/27)	36.4% (16/44)	28.1% (93/331)	32.7% (270/825)
Low dose (<0.9 mg/kg)	72.8% (155/213)	70.3% (26/37)	74.1% (20/27)	63.6% (28/44)	71.9% (238/331)	67.3% (555/825)
Onset to needle time (min)	121.0 ± 59.8	130.5 ± 50.0	123.1 ± 55.8	105.0 ± 54.9	119.8 ± 58.3	120.8 ± 56.6

*AC, anticoagulant; AP, antiplatelet; ASA, aspirin; aPTT, activated partial thromboplastin time; BP, blood pressure; DAPP, dual antiplatelet pre-treatment of aspirin and clopidogrel; INR, international normalized ratio; NIHSS, the National Institute of Health Stroke Scale; P2Y12, purinergic receptor P2Y12 inhibitor of clopidogrel and ticlopidine; WAR, warfarin*.

*P-values indicate comparison of each AP/AC vs. No AP/AC (Student's t-test for continuous variable and Pearson Chi-squared test for categorical variables)*.

* *P < 0.05*,

** *P < 0.01*,

*** *P < 0.001*.

### Distribution of Global Functional Outcomes by Dosage of Alteplase

**Figure 1:**
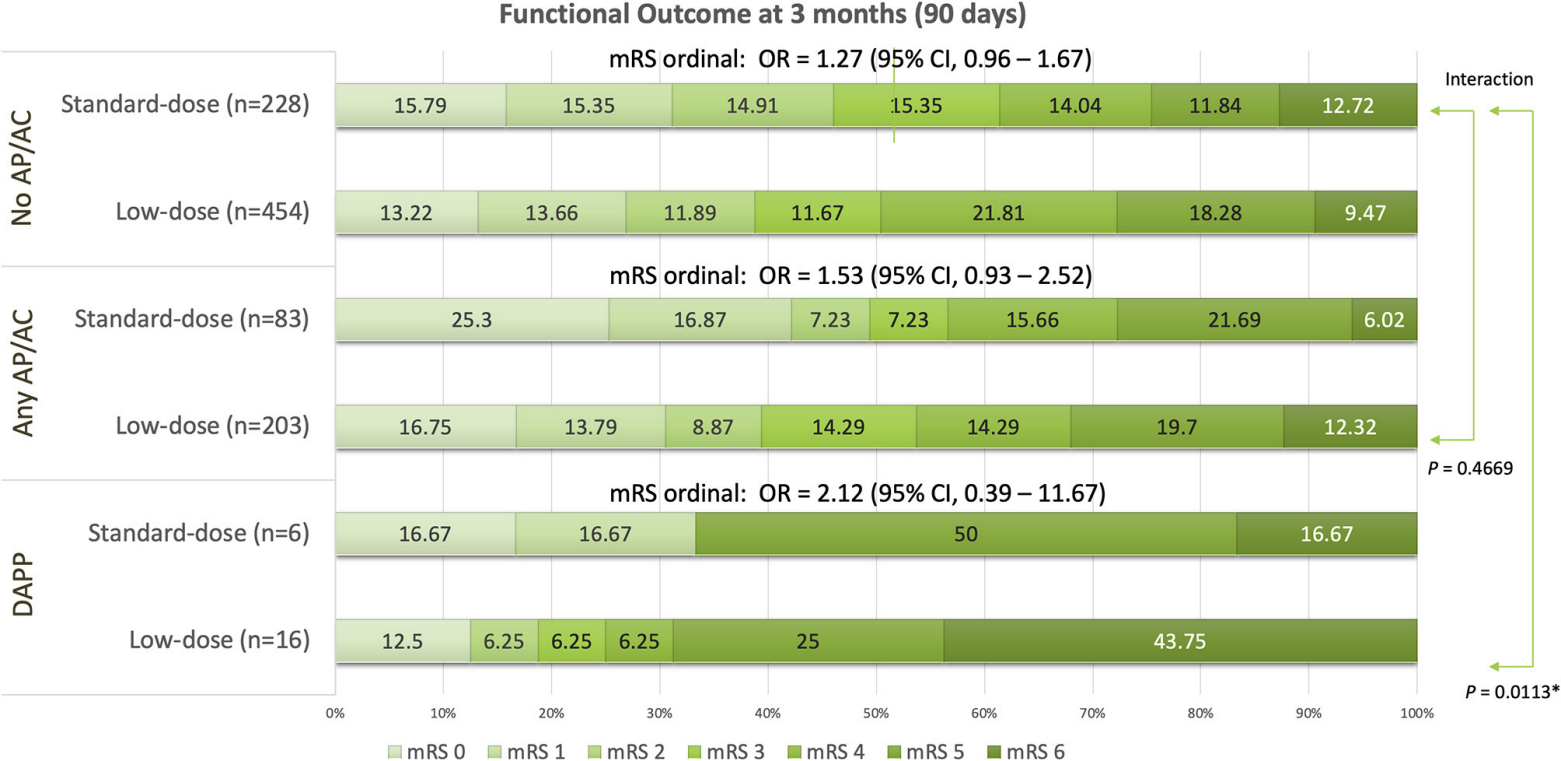
Global functional outcomes at 90 days for groups of patients having pre-treatment without any antiplatelets or anticoagulants (no AP/AC), with any antiplatelets or anticoagulant (any AP/AC), and with dual antiplatelet (DAPP). *P < 0.05.

Global function outcomes for the three groups of no AP/AC, any AP/AC, and DAPP are shown in Figure 1. Compared to standard-dose alteplase, low-dose alteplase presented no significant increase in the ordinal mRS for no AP/AC (OR: 1.27, 95% CI, 0.96–1.67), for any AP/AC (OR: 1.53, 95% CI, 0.93–2.52), and for the DAPP group (OR: 2.12, 95% CI, 0.39–11.67), respectively. However, a significant interaction was found between the dosage of alteplase and the use of DAPP (*P* = 0.0113). In the DAPP group, low-dose alteplase had a higher proportion of unfavorable functional outcomes and death.

### Outcome Measures of Safety

The outcome measures for each pre-treatment of the AP/AC groups are shown in Table 2. Except for the DAPP group, the cumulative incidence of SICH was ~2–5% by the NINDS and 1–3% by the ECASS II standards for each group, respectively. The DAPP group showed an extremely high cumulative incidence of SICH (11.1 and 7.4% according to the NINDS and ECASS II criteria, respectively). Pre-treatment with single antiplatelet agents of ASA or P2Y12 or anticoagulant of warfarin consistently showed no significant increase in SICH in both the simple and multivariate logistic regression models. In contrast, the DAPP group exhibited a significantly higher risk of SICH by the NINDS (OR: 5.61, 95% CI, 1.55–20.32, adjusted OR: 4.90, 95% CI, 1.28–18.69 in the adjusted model) and the ECASS II standards (OR: 5.61, 95% CI, 1.55–20.32 and adjusted OR: 4.90, 95% CI, 1.28–18.69), respectively. Of the 10 patients with other antiplatelets, no patients developed SICH (supplementary Table 2).

**Table 2:** Functional Outcome at 3 months (3 m).

**Functional outcomes**	**ASA (*n* = 188)**	**P2Y12 (*n* = 32)**	**DAPP (*n* = 22)**	**WAR (*n* = 36)**	**Any AP/ AC (*n* = 286)**	**No AP/ AC (*n* = 682)**
**SYMPTOMATIC INTRACRANIAL HEMORRHAGE (SICH)**						
**SICH per NINDS**						
*n*/ total *n* (%)	3.3% (7/213)	5.4% (2/37)	11.1% (3/27)	2.3% (1/44)	3.9% (13/331)	2.2% (25/1,139)
OR (95% CI)	1.52 (0.63–3.70)	2.56 (0.57–11.48)	5.61 (1.55–20.32)^**^	1.04 (0.14–7.99)	1.83 (0.89–3.79)	Ref. group
Adjusted OR^†^	0.99 (0.38–2.59)	1.95 (0.43–8.84)	4.90 (1.28–18.69)^*^	0.83 (0.19–6.94)	1.34 (0.62–2.90)	Ref. group
**SICH per ECASS II**						
*n*/total *n* (%)	2.8% (6/213)	2.7% (1/37)	7.4% (2/27)	2.3% (1/44)	3.0% (10/331)	1.3% (11/825)
OR (95% CI)	2.15 (0.78–5.87)	2.06 (0.26–16.36)	5.92 (1.25–28.13)^*^	1.72 (0.22–13.64)	2.31(0.97–5.48)	Ref. group
Adjusted OR^†^	1.38 (0.46–4.16)	1.68 (0.21–13.69)	5.09 (1.01–25.68)^*^	1.97 (0.21–18.75)	1.70(0.71–4.51)	Ref. group
**FAVORABLE FUNCTIONAL OUTCOME (FFO)**						
**mRS of 0–1 at 90 days**						
*n*/ total *n* (%)	38.3% (72/188)	28.1% (9/32)	18.2% (4/22)	30.6% (11/36)	33.9% (97/286)	28.3% (193/682)
OR (95% CI)	1.57 (1.12–2.21)^**^	0.99 (0.45–2.18)	0.56 (0.19–1.69)	1.12 (0.54–2.31)	1.30 (0.97–1.75)	Ref. group
Adjusted OR^†^	1.91 (1.31–2.78)^***^	1.20 (0.51–2.82)	0.66 (0.21–2.06)	1.03 (0.43–2.45)	1.51 (1.08–2.10)	Ref. group
**FUNCTIONAL INDEPENDENCE (FI)**						
**mRS of 0–2 at 90 days**						
*n*/total *n* (%)	45.2% (85/188)	34.4% (11/32)	22.7% (17/22)	50.0% (18/36)	42.3% (121/286)	41.2% (281/682)
OR (95% CI)	1.18 (0.85–1.63)	0.75 (0.36–1.58)	0.42 (0.15–1.15)	1.43 (0.73–2.79)	1.05 (0.79–1.38)	Ref. group
Adjusted OR^†^	1.39 (0.97–1.99)	0.94 (0.42–2.08)	0.54 (0.19–1.55)	1.59 (0.75–3.39)	1.19 (0.87–1.62)	Ref. group
**Death at 90 days**						
*n*/ total *n* (%)	7.5% (14/188)	15.6% (5/32)	36.4% (8/22)	8.3% (3/36)	10.5% (30/286)	10.6% (72/682)
OR (95% CI)	0.68 (0.38–1.24)	1.57 (0.59–4.20)	4.84 (1.96–11.94)^***^	0.77 (0.23–2.58)	0.99 (0.63–1.56)	Ref. group
Adjusted OR^†^	0.68 (0.36–1.29)	1.92 (0.70–5.32)	4.75 (1.77–12.72)^**^	0.83 (0.24–2.90)	0.99 (0.61–1.61)	Ref. group

*AC, anticoagulant; AP, antiplatelet; ASA, aspirin; DAPP, dual antiplatelet pre-treatment of aspirin and clopidogrel; ECASS II, the European-Australasian Acute Stroke Study II; FFO, favorable functional outcome; FI, functional independence; mRS, modified Rankin Scale; P2Y12, purinergic receptor P2Y12 inhibitor of clopidogrel and ticlopidine; NINDS, National Institute of Neurological Disorders and Stroke Study; SICH, symptomatic intracranial hemorrhage; War, warfarin; mRS, modified Rankin Scale; OR, odds ratio*.

* *P < 0.05*,

** *P < 0.01*,

*** *P < 0.001*.

† *Model adjusted for hypertension, diabetes, hyperlipidemia, atrial fibrillation, and alteplase dose*.

### Outcome Measures of Efficacy

For outcomes of FFO of mRS of 0–1, the ASA group showed a greater number of patients with better functional outcome (38.3%) and the DAPP group showed a lower number of patients (18.2%). Among pre-treatment groups, the ASA group was the only one that showed significant improvements of FI (OR: 1.57, 95% CI, 1.12–2.21, and adjusted OR: 1.91, 95% CI, 1.31–2.78), respectively. Despite the DAPP group with a lower proportion of FFO, no significant difference was observed in the outcomes of FFO in comparison to the no AP/AC group. As for the FI of mRS of 0–2, the trend was similar to that of FFO, but no significant difference was found among pre-treatment groups. For outcome of mortality, each group had <10% mortality, except for the P2Y12 (15.6%) and DAPP groups (36.4%). Of them, the DAPP group showed a significant 4- to 5-fold risk of mortality (OR: 4.84, 95% CI, 1.96–11.94; adjusted OR: 4.75, 95% CI, 1.77–12.72).

## Discussion

In this study, we found a significant interaction between the dosage of alteplase and DAPP for the global functional outcome. In the DAPP group, low-dose alteplase had a higher proportion of unfavorable outcomes despite no significantly increased ordinal mRS. For the older adults, pre-treatment with ASA resulted in significant improvement of FFO but not FI. The DAPP group had lower proportions of better outcomes, but no significant difference in terms of FFO and FI compared to no AP/AC. However, DAPP was found to have a significantly increased 5-fold the risk of SICH with both NINDS and ECASS II standards. Mortality was found to be significantly increased by more than 4-fold in the DAPP group.

Our analysis was in line with most of the observational studies, which showed that prior dual antiplatelets increased the 4- to 9-fold risk of SICH (4–6). In contrast, our results showed some disagreement from two recent studies employing propensity score matching (PSM) adjustment (7, 8), which found that DAPP did not significantly increase SICH after adjustment for confounders. In fact, these studies employing PSM adjustment enrolled patients with mild severity of acute ischemic stroke (average NIHSS score of <10) (7, 8), while we included patients with higher severity (mean NIHSS score of 13–16). In addition, the control group showed different characteristics between these studies and ours (8). Their reference group included patients pre-treated with a single antiplatelet agent and no antiplatelets, whereas our reference group was treated without any AP/AC. Moreover, our patients were older. These characteristic differences between our enrolled patients and theirs, should cause the diverged results. On the other hand, the WAR group in our study showed no significant difference in comparison to no AP/AC. Since patients treated with warfarin with INR > 1.7 were excluded for IVT in our protocol, patients in the WAR group actually were below of their therapeutic range. This should explain the lower SICH rates in the WAR group.

Besides, this study revealed the real-world dosing patterns of IVT for eligible patients with acute ischemic stroke in Taiwan and other east Asian countries. In 2006, the Japan Alteplase Clinical Trial (J-ACT) showed equivalent efficacy and higher safety results of IV thrombolysis using alteplase at a dose of 0.6 mg/kg (16). Thereafter, the Japanese drug safety authority approved the dose. In 2010, an observational study in Taiwan (TTT-AIS) showed the standard dose of 0.9 mg/kg alteplase may not be optimal for aged population (11). In 2014, another study in Taiwan (TTT-AIS II) showed a lower dose of 0.6 mg/kg was associated with a better outcome for elderly (age group of 71–80 years) as well (12). In the background, most of the neurologists in Taiwan tended to adopt a lower dose of alteplase for IV thrombolysis during our enrollment. On the other hand, in 2018, an another analysis for Taiwanese octogenarian stroke patients with higher severity (high NIHSS score of ≥ 14 at initial presentation) showed a standard dose of 0.9 mg/kg with higher rates of the FFO in comparison to lower dose of 0.6 mg/kg (17). For mild stroke (NIHSS score of 4–8), both standard-dose and low-dose alteplase showed comparable rates of favorable functional outcomes, but low-dose alteplase for mild stroke showed much reduced mortality on day 90 for octogenarians (17). In our study, the stroke patient characteristics (Table 1) showed higher baseline severity (mean NIHSS score of 13–15 for all pre-treatment groups). We considered a lower dose should be inadequate for the stroke patients with higher severity patients and the results showed higher death rates.

Our inclusion of stroke patients of >60 years for IVT should be representative of the majority stroke patients in Taiwan. The demographic data in Taiwan showed the incidence rates of first stroke for age below 60 years were extremely low (18, 19). Among the largest cohort of 8,562 stroke-free people in Taiwan followed for 4 years, the incidence rate of first stroke for age groups of 36–44 and 45–54 years were 2 per 100,000 and 12 per 100,000 person-years, respectively (18). The incidence rate of first stroke throughout all age groups was 104 per 100,000 person-years (18). In contrast, the incidence rate of first stroke for age groups of 55–64, 65–74, and over 75 years were, respectively, 41 per 100,000, 29 per 100,000 and 20 per 100,000 years (18). The proportion of first stroke incidence rate for age below 55 years in Taiwanese population was 13.5%.

The propensity score matching was not conducted in our study due to our multiple pre-treatment groups (five groups). Currently, methods of binary treatment (two groups) propensity scoring are well-developed and established completely (20–22). Methods of multinomial treatment (>2 groups) propensity scoring have been in the developing status and were increasing unstable with expanding the comparison groups since the current distance-based matching approach cannot be extended to more than three groups (21, 23–25). Since we had five pre-treatment groups of ASA, P2Y12, DAPP, WAR, and any AP/AC, using the method of binary treatment propensity score matching would produce five reference groups of No AP/AC. The demographic and characteristic composition among the new five reference groups for those ASA, P2Y12, DAPP, WAR, and any AP/AC were totally different. The efficacy and safety outcome among the five pre-treatment groups were not comparable. To reasonably comparing with the five pre-treatment groups, we preferred to use method of multivariate logistic regression since they shared the same reference group.

Our studies have robust strengths and offer distinctive information. First, the patients enrolled were homogenous for the baseline demographic and medical characteristics among the six groups, such as age, sex, laboratory tests, alteplase dose, and the onset to needle time. Second, patients who were enrolled in the study had moderate to high baseline severity of acute ischemic stroke. Third, we found significant interactions between different doses of alteplase and DAPP. Fourth, we focused on older patients. This should offer the evidence that DAPP should still be cautious for older adults with acute ischemic stroke treated with IVT. Lastly, TTT-AIS was a multicenter study across all regions in Taiwan, and the representative nationwide cohort was used for analysis (11–13). This study investigated the efficacy and safety of antithrombotic pre-treatment in real-life study model.

This study has some limitations. First, the prospective cohort study design in our study was still susceptible to residual confounding. Some unmeasured confounding effects were not controlled. In addition, the analysis in this study excluded patients aged <60 years, and these findings may not be applicable throughout all ages. Second, there were a small number of patients in the DAPP group (2.3%, *n* = 27) and therefore the outcomes of SICH per NINDS (three out of 27 patients, adjusted OR: 4.90, 95% CI 1.28–18.69) and per ECASS II (two out of 27 patients, adjusted OR: 5.09, 95% CI: 1.01–25.68) in the DAPP group showed wide confidence interval. Although the number of DAPP patients in this study was small, this low proportion reflects real-world conditions. Previous observational studies indicated that the proportion of DAPP ranged from 1.3 to 7.3% for all ischemic stroke patients treated with IVT. Third, warfarin group was not representative to those patients treated with sufficient dose of warfarin. Only stroke patients insufficiently treated with warfarin (INR <1.7) and IVT were included for this analysis. Lastly, some patients had incomplete follow-up at 90 days. In Taiwan, the prolonged hospital stay and readmission for stroke patients are serious problems. Since March 2014, the nationwide post-acute stroke care (PAC) program (26) was launched to improve the problems of shortage of acute beds, and overcrowded emergency departments. Stroke patients with stable neurological functional status for ≥72 h and no uncontrolled complications were transferred to regional hospitals. Some patients participating in the PAC program were unwilling to be contacted. While the follow-up rates for groups of ASA, P2Y12, DAPP, and WAR groups were 88.3% (188/213), 86.5% (32/37), 81.5% (22/27), and 81.8% (36/44), the follow-rate for no AP/AC group was 82.7% (682/825). Theoretically, no differential incomplete follow-up (27) were found between groups with and without AP/AC pre-treatment, and the validity in our analysis was still assured.

In clinical practice, physicians should be cautious for older patients receiving DAPP before IVT. Since a significant interaction for functional outcome was noted between the different dosage of alteplase and use of DAPP (Figure 1), standard-dose alteplase still could be considered for these stroke patients with high-risk and higher baseline severity. In our previous analysis for 249 old stroke patients over the age of 80 years, standard-dose alteplase was associated with an increased proportion of FI of mRS 0–2 (34.8 vs. 22.2%) and a little increased mortality (13.5 vs. 9.3%) at 90 days (17). Nevertheless, the previous subgroup analysis for a total of 128 octogenarian patients of high severity (NIHSS ≥ 14) showed an increased proportion of FI of mRS 0–2 (20.8 vs. 8.9%) and a near equivalent mortality (15.3 vs. 14.3%) (17). Although an early observational study of IVT in Japan showed similar functional outcomes and approved a low-dose IVT of 0.6 mg/kg (16), we did not recommend universal use of low-dose IVT for all older patients. Consequently, physicians should evaluate many factors of age, baseline stroke severity, comorbidities, and pre-treatment of antiplatelets and anticoagulants when prescribing the optimal dose of alteplase for acute ischemic stroke patients.

## Conclusion

In conclusion, pre-treatment with ASA seems to improve functional outcomes in terms of FFO (mRS of 0–1), but not of FI (mRS 0–2). For older adults, DAPP increases the risk of SICH, especially for patients presenting themselves with moderate to high severity of acute ischemic stroke following IVT. In addition, DAPP increased the risk of mortality for older adults and showed no increase for the better outcomes in terms of FFO and FI. Nevertheless, DAPP still should not be the reason to hold IVT and to prescribe low-dose IVT in our analysis.

## Data Availability Statement

The datasets presented in this article are not readily available because distribution of dataset was restricted by Institutional Review Board of Kaohsiung Medical University. Requests to access the datasets should be directed to A-Ching Chao, achch@cc.kmu.edu.tw.

## Ethics Statement

The studies involving human participants were reviewed and approved by Institutional Review Board of Kaohsiung Medical University. The patients/participants provided their written informed consent to participate in this study.

## Author Contributions

S-FL wrote the first draft of the manuscript. All authors contributed to the conception and design of the study, acquisition, analysis, and interpretation of data.

## Conflict of Interest

The authors declare that the research was conducted in the absence of any commercial or financial relationships that could be construed as a potential conflict of interest.
