# Corrigendum: Pre-treatment of Single and Double Antiplatelet and Anticoagulant With Intravenous Thrombolysis for Older Adults With Acute Ischemic Stroke: The TTT-AIS Experience

**DOI:** 10.3389/fneur.2021.737437

**Published:** 2021-09-17

**Authors:** Sheng-Feng Lin, Han-Hwa Hu, Bo-Lin Ho, Chih-Hung Chen, Lung Chan, Huey-Juan Lin, Yu Sun, Yung-Yang Lin, Po-Lin Chen, Shinn-Kuang Lin, Cheng-Yu Wei, Yu-Te Lin, Jiunn-Tay Lee, A-Ching Chao, Taiwan Thrombolytic Therapy for Acute Ischemic Stroke (TTT-AIS) Study Group

**Affiliations:** ^1^School of Public Health, College of Public Health, Taipei Medical University, Taipei, Taiwan; ^2^Department of Critical Care Medicine, Taipei Medical University Hospital, Taipei, Taiwan; ^3^Department of Clinical Pathology, Taipei Medical University, Taipei, Taiwan; ^4^Beijing Tiantan Hospital, Capital Medical University, Beijing, China; Advanced Innovation Center for Human Brain Protection, Capital Medical University, Beijing, China; ^5^Department of Neurology, Taipei Medical University-Shaung Ho Hospital, Taipei, Taiwan; ^6^Department of Neurology, Kaohsiung Medical University Hospital, Kaohsiung, Taiwan; ^7^Department of Neurology, Kaohsiung Medical University, Kaohsiung, Taiwan; ^8^Department of Neurology, National Cheng Kung University Hospital, Tainan, Taiwan; ^9^Department of Neurology, National Cheng Kung University, Tainan, Taiwan; ^10^Department of Neurology, Chi Mei Medical Center, Tainan, Taiwan; ^11^Department of Neurology, En Chu Kong Hospital, New Taipei City, Taiwan; ^12^Department of Neurology, Taipei Veterans General Hospital, Taipei, Taiwan; ^13^Department of Neurology, Taichung Veterans General Hospital, Taichung, Taiwan; ^14^Stroke Center and Department of Neurology, Taipei Tzu Chi Hospital, Buddhist Tzu Chi Medical Foundation, Taipei, Taiwan; ^15^Department of Neurology, Show Chwan Memorial Hospital, Changhua, Taiwan; ^16^Division of Neurology, Department of Medicine, Kaohsiung Veterans General Hospital, Kaohsiung, Taiwan; ^17^Department of Neurology, National Defense Medical Center, Tri-Service General Hospital, Taipei, Taiwan

**Keywords:** aspirin, clopidogrel, stroke, intracranial hemorrhage, intravenous thrombolysis

In the published article, there were errors in affiliations 1–5. Instead of “1 School of Public Health, College of Public Health, Taipei, Taiwan, 2 Beijing Tiantan Hospital, Capital Medical University, Beijing, China, 3 Advanced Innovation Center for Human Brain Protection, Capital Medical University, Beijing, China, 4 Department of Neurology, Taipei Medical University-Shaung Ho Hospital, Taipei, Taiwan, 5 Department of Health Care Administration, Chung-Hwa University of Medical Technology, Tainan, Taiwan” it should be “1 School of Public Health, College of Public Health, Taipei Medical University, Taipei, Taiwan, 2 Department of Critical Care Medicine, Taipei Medical University Hospital, Taipei, Taiwan, 3 Department of Clinical Pathology, Taipei Medical University, Taipei, Taiwan, 4 Beijing Tiantan Hospital, Capital Medical University, Beijing, China; Advanced Innovation Center for Human Brain Protection, Capital Medical University, Beijing, China, 5 Department of Neurology, Taipei Medical University-Shaung Ho Hospital, Taipei, Taiwan.”

The authors apologize for this error and state that this does not change the scientific conclusions of the article in any way. The original article has been updated.

## Publisher's Note

All claims expressed in this article are solely those of the authors and do not necessarily represent those of their affiliated organizations, or those of the publisher, the editors and the reviewers. Any product that may be evaluated in this article, or claim that may be made by its manufacturer, is not guaranteed or endorsed by the publisher.

