# Supplementary material for: Pre-treatment of Single and Double Antiplatelet and Anticoagulant With Intravenous Thrombolysis for Older Adults With Acute Ischemic Stroke: The TTT-AIS Experience
Source: Front Neurol. 2021 Feb 22;12:628077. doi: 10.3389/fneur.2021.628077 (PMC7937707; doi:10.3389/fneur.2021.628077)
Supplement: Supplementary file 1 [file Data_Sheet_1.PDF]

Supplemental Table 1. Characteristics of Patients Receiving Antiplatelets of Dipyridamole and Cilostazol Before Intravenous Thrombolysis (Total $N = 10$)

	Dipyridamole (n=4)	Cilostazol (n=6)	Other AP (n=10)
Age (years)	68.0 ± 5.1	76.3 ± 12.3	73.0 ± 10.6
Age groups (years)			
60–69 years	75.0% (3/4)	33.3% (2/6)	50.0% (5/10)
70–79 years	25.0% (1/4)	33.3% (2/6)	30.0% (3/10)
80–89 years	0% (0/4)	16.7% (1/6)	10.0% (1/10)
≥90 years	0% (0/4)	16.7% (1/6)	10.0% (1/10)
Female sex (%)	25.0% (1/4)	66.7% (4/6)	50.0% (5/10)
Comorbidity (%)			
Hypertension	75.0% (3/4)	83.3% (5/6)	80.0% (8/10)
Diabetes	50.0% (2/4)	50.0% (3/6)	50.0% (5/10)
Hyperlipidemia	25.0% (1/4)	16.7% (1/6)	20.0% (2/10)
Atrial fibrillation	0% (0/2)	33.3% (2/6)	25.0% (2/8)
Alcoholism	0% (0/4)	16.7% (1/6)	10.0% (1/10)
Glucose (mg/dl)	231.0 ± 124.5	124.8 ± 15.3	151.4 ± 69.2
Prothrombin time (INR)	1.00 ± 0.00	1.00 ± 0.06	1.00 ± 0.05
aPTT	25.6 ± 3.3	27.3 ± 1.7	26.8 ± 2.1
Systolic BP (mmHg)	158.0 ± 7.1	145.2 ± 27.2	148.4 ± 23.9
Diastolic BP (mmHg)	98.0 ± 7.1	83.0 ± 10.2	86.8 ± 11.4
Baseline NIHSS	11.0 ± 6.4	15.5 ± 9.4	13.7 ± 8.3
Alteplase dose (mg/kg)	0.75 ± 0.10	0.73 ± 0.12	0.74 ± 0.11
Standard dose (0.9 mg/kg)	25.0% (1/4)	0% (0/6)	10.0% (1/10)
Low dose (<0.9 mg/kg)	75.0% (3/4)	100.0% (6/6)	90.0% (9/10)
Onset to needle time (min)	72.5 ± 84.6	138.7 ± 52.4	112.2 ± 71.3

Abbreviations: AP, antiplatelet; aPTT, activated partial thromboplastin time; BP, blood pressure; INR, international normalized ratio; NIHSS, the National Institute of Health Stroke Scale

Table 2. Functional Outcome at 3 months (3m).

Functional outcomes	Dipyridamole (n=2)	Cilostazol (n=6)	Other AP (n=8)
SICH per NINDS (%)	0% (0/2)	0% (0/6)	0% (0/10)
SICH per ECASS II (%)	0% (0/2)	0% (0/6)	0% (0/10)
mRS of 0–1 at 90 days (%)	0% (0/2)	16.7% (1/6)	12.5% (1/8)
mRS of 0–2 at 90 days (%)	50.0% (1/2)	16.7% (1/6)	25.0% (2/8)
Death at 90 days (%)	0% (0/2)	0% (0/6)	0% (0/8)

Abbreviations: AP, antiplatelet; ECASS II, the European-Australasian Acute Stroke Study II; mRS, modified Rankin Scale; NINDS, National Institute of Neurological Disorders and Stroke Study; SICH, symptomatic intracranial hemorrhage
